# Providing immediate neonatal care and resuscitation at birth beside the mother: clinicians’ views, a qualitative study

**DOI:** 10.1136/bmjopen-2015-008494

**Published:** 2015-09-30

**Authors:** Charles W Yoxall, Susan Ayers, Alexandra Sawyer, Sophia Bertullies, Margaret Thomas, Andrew D Weeks, Lelia Duley

**Affiliations:** 1 Neonatal Unit, Liverpool Women's Hospital, Liverpool, UK; 2 Centre for Maternal and Child Health Research, City University London, London, UK; 3 Department of Women's and Children's Health, University of Liverpool, Liverpool, UK; 4 Nottingham Clinical Trials Unit, University of Nottingham, Nottingham, UK

**Keywords:** NEONATOLOGY, QUALITATIVE RESEARCH

## Abstract

**Objectives:**

The aims of this study were to assess clinicians’ views and experiences of providing immediate neonatal care at birth beside the mother, and of using a mobile trolley designed to facilitate this bedside care.

**Design:**

Qualitative interview study with semistructured interviews.

**Results:**

The results were analysed using thematic analysis.

**Setting:**

A large UK maternity unit.

**Participants:**

Clinicians (n=20) from a range of disciplines who were present when the trolley was used to provide neonatal care at birth at the bedside. Five clinicians provided/observed advanced resuscitation by the bedside.

**Results:**

Five themes were identified: (1) Parents’ involvement, which included ‘Contact and involvement’, ‘Positive emotions for parents’ and ‘Staff communication’; (2) Reservations about neonatal care at birth beside the mother, which included ‘Impact on clinicians’ and ‘Impact on parents’; (3) Practical challenges in providing neonatal care at the bedside, which included ‘Cord length’ and ‘Caesarean section’; (4) Comparison of the trolley with usual resuscitation equipment and (5) Training and integration of bedside care into clinical routine, which included ‘Teething problems’ and ‘Training’.

**Conclusions:**

Overall, most clinicians were positive about providing immediate neonatal care at the maternal bedside, particularly in terms of the clinicians’ perceptions of the parents’ experience. Clinicians also perceived that their close proximity to parents improved communication. However, there was some concern about performing more intensive interventions in front of parents. Providing immediate neonatal care and resuscitation at the bedside requires staff training and support.

**Strengths and limitations of this study**

- This is the first in-depth study of clinicians’ experiences of immediate newborn care and resuscitation at the maternal bedside. Use of in-depth qualitative methods allowed a detailed exploration of these experiences.
- Healthcare professionals with a wide range of clinical backgrounds and experience were interviewed.
- A limitation of the study is that clinicians were recruited from a single site; the site had pioneered this type of care.

## Introduction

In the UK, approximately 30% of births are attended by a clinician trained in newborn life support, although only a minority of infants will require immediate resuscitation.^1^ Usual practice of clinicians providing care for infants requiring care at birth is that, following immediate cord clamping and cutting, the baby is taken away from the mother for initial assessment and stabilisation. This takes place on a resuscitation platform and overhead warmer that is usually located at the side of the room, or sometimes in another room. With this equipment against the wall, the neonatal team will have their backs to the mother and her birth partner, who are therefore shielded from their baby.^2–4^ The importance of families being present during resuscitation of adults and children is well established, and is now standard care.^5–10^ Clinicians have mixed opinions about family presence during resuscitation, with those less experienced more likely to have reservations.^10–12^ Only one study that explored clinicians’ experiences of newborn resuscitation at birth in front of parents focused only on the father's presence and found that some healthcare professionals felt uncomfortable when fathers witnessed the resuscitation.^12^

As part of a programme of work to improve outcome and quality of care following preterm birth, we developed strategies for providing immediate neonatal care at birth beside the mother. The aims were to allow parents to share the first moments of their baby's life and, as part of planning a randomised trial, of deferring cord clamping for very preterm births,^13^ and to assess whether it is possible to provide newborn life support with the umbilical cord intact. We have shown that it is possible to successfully provide immediate neonatal care beside the mother either with a mobile trolley designed for this purpose (the Bedside Assessment, Stabilisation and Initial Cardiorespiratory Support (BASICS) trolley, marketed as LifeStart),^14^ ^15^ or by moving and adjusting the standard resuscitation equipment.^16^ The aim of this study was to assess clinicians’ views of providing neonatal care and resuscitation at birth beside the mother, and to document their experiences of using the trolley. Views and experiences of parents are reported in a separate paper.^17^

## Methods

Recruitment was between November 2012 and January 2014, in a large maternity hospital where, to provide neonatal care beside the mother, use of the trolley was introduced into clinical service in November 2012. For this qualitative study, purposive sampling was used where clinicians who were present when the trolley was used were invited to participate. All participants were approached in person and provided with written information. Written informed consent was obtained from each participant. Interviews were carried out by a female psychologist (PhD) or by one of the three female research midwives trained in qualitative methods. The interviewers are not authors on this paper. The three research midwives who conducted the interviews worked in the hospital where the trolley was developed. However, they were not involved in the design of the trolley. As midwives, they did have opinions about the possible advantages of providing care by the mother's bedside. The psychologist who also conducted the interviews had no experience of perinatal psychology and held no views about the advantages of bedside care. None of the interviewers were involved in the mother's or baby's care, however, the research midwives were colleagues of the staff interviewed.

Three of the authors (LD, CWY and ADW) were involved in the initial design of the trolley and are aware of the potential benefits of providing immediate neonatal bedside care beside the mother. MT is a research midwife who also works at the hospital where the trolley was developed and has been involved in a previous questionnaire study of clinicians’ experiences of the trolley, which reported positive preliminary findings. AS and SA are psychologists with significant experience in the area of maternal and child health. Although they were not involved in the design of the trolley and are not clinicians, they are aware of the importance of the birth experience for parents. AS and SA have also both been involved throughout the project and therefore have significant background knowledge of the trolley and potential advantages of bedside care. However, although holding positive views on bedside care, these six authors were not involved in data collection or initial analysis of the interviews. SB is a Masters level psychologist and was not involved in any part of the programme prior to working on the data analysis for this study. Therefore, when it came to analysing the interviews, she did not have any prior assumptions about the trolley and neonatal bedside care.

The respective interviewer would introduce herself and explain the purpose of the research. Interviews lasted approximately 20 min, and were audio recorded and transcribed with all identifying information removed. Data collection ended when data saturation had been achieved. This was when no new information in relation to the study aims and interview questions were emerging from the interviews.

The interview schedule consisted of open-ended questions, which were used to explore clinicians’ views and experiences of providing neonatal care at the bedside, and of the trolley (see online supplementary Appendix A). Interviews were conducted in a private office in the neonatal unit. No one was present apart from the interviewer and interviewee. The interviewer had the freedom to probe the interviewee to elaborate responses or follow a line of inquiry introduced by the interviewee. Cues and prompts were also used by the researcher to allow the interviewee to discuss the topic further.

### Data analysis

Qualitative analysis of the transcripts used inductive thematic analysis to identify, describe and analyse themes and patterns within the data.^17^ First, transcripts were read and reread to familiarise the researchers (AS and SB) with the data. Second, all interviews were coded in detail to ensure all codes arising were included in an initial pool of codes. Third, the pool of codes was sorted into potential themes on the basis of frequency, significance and overlap. Where there was overlap between codes, these were collated into themes or subthemes. Fourth, themes were reviewed by three authors (AS, SA and SB) in relation to the generated codes and the entire data set. Finally, themes were named and defined in a coding schedule, which was used to code all interviews again to ensure reliability and consistency of coding. NVivo V.10 software (QSR International Pty Ltd) was used to organise codes and themes.

## Results

Twenty-six clinicians were approached. All initially consented to be interviewed, but six later declined, leaving 20 clinicians interviewed (see table 1). Five clinicians provided/observed advanced resuscitation at the bedside. Overall experiences of bedside neonatal care were mostly positive or conditionally positive (see table 2). Five themes were identified: (1) Parents’ involvement; (2) Reservations about neonatal care at birth beside the mother; (3) Practical challenges in providing neonatal care at the bedside; (4) Comparison of the trolley with usual resuscitation equipment and (5) Training and integration of bedside care into clinical routine. These are described in more detail below and illustrated by the quotes in table 3.

**Table 1:** Characteristics of clinicians interviewed

	N=20 (%)
Professional role	
Advanced neonatal practitioner	10 (50)
Consultant obstetrician	1 (5)
Consultant neonatologist	2 (10)
Neonatal nurse	1 (5)
Senior house officer: paediatrics	4 (20)
Midwife	1 (5)
Senior registrar: paediatrics	1 (5)
Role in providing neonatal care at birth	
Observed	2 (10)
Participated	18 (90)
Trolley experience*	
Used once	9 (50)
Used more than once	9 (50)

*Owing to missing data, n=18.

**Table 2:** Overall evaluation of trolley given by clinicians (N=18)

Positive evaluation	Mixed/conditionally positive evaluation	Negative evaluation
5/10 ANNPs	4/10 ANNPs	1/10 ANNP
2/2 Consultant neonatologists	3/4 SHOs	
1/1 Neonatal nurse	1/1 Midwife	
	1/1 Consultant obstetrician	

18/20 Clinicians gave an overall evaluation of the trolley. One Paediatric Senior Registrar was also interviewed but did not give an overall evaluation.

ANNP, advanced neonatal practitioner; SHOs, senior house officers.

**Table 3:** Themes, subthemes and sample quotes from the 20 interviews with clinicians

Theme/subtheme	Sample quotes	Number of interviews themes/subthemes mentioned in
1. Parents’ involvement		
Contact and involvement	“[…] if it is on that trolley, by the bed, they can at least see what is being done for the baby and what is going on.” (*21; midwife*) “Even the babies that are just 32 weekers/33 weekers who won't necessarily need resuscitation but it just means that they're there with mum before whizzing them away.” (*4; ANNP*) “Because you know, I think for the mother a C-section is not what you've planned for […] so at least you are with the baby which is more natural. More natural in an unnatural way.” (*3; ANNP*) “I think because it was a section the screen was up, so they obviously couldn't see it happening.” (*10; neonatal nurse*)	18
Positive emotions for parents	“So for that I think it's perfect, because mum actually did say that she like the fact that she could see the baby and touch the baby.” (*12; SHO*)	8
Staff communication	“I found the big positive was that you weren't far away in a corner and so you immediately started to talk to the parents about the baby.” (*15; consultant neonatologist*) “It's easier to say while you are there ‘Oh we're just giving a few breaths’ rather than shout across the room.” (*16; SHO*) “Massive difference [between practitioners] and […] having a trolley shouldn't make you interact with parents because you are near somebody's legs.” (*24; ANNP*)	8
2. Reservations about neonatal care at birth beside the mother		
Impact on clinicians	“It doesn't bother me in the slightest.” (*9; ANNP*) “Although, the worry for junior doctors, which is why some people don't like it, is not actually the equipment itself, it's more that you feel that you are on show. Your skills are going to be judged.” (*12; SHO*) “You're then sort of setting a precedent for ‘OK, well we'll do this in front of your child’ and then it's when they're on intensive care, and they then expect, which, you know, it might be fine, their baby's…their LPs to be done in front of them and their intubation and everything else.” (*15; consultant neonatologist*)	16
Impact on parents	“I guess, we sort of thought, when we left, we also discussed and said ‘well, would that be traumatic for them to see, or would it be beneficial?’ And we actually felt it would be quite traumatic for them, the fact that they had to do chest compressions, but I understand that afterwards, they didn't find it traumatic.” (*15; consultant neonatologist*)	12
3. Practical challenges in providing neonatal care at the bedside		
Cord length	“I think, again, the one in theatre when we used it was quite a short cord, so it was difficult and they couldn't do the delayed cord clamping because it just wouldn't reach.” (*19; SHO*)	10
Caesarean section	“I think because it was a section the screen was up, so they obviously couldn't see it happening.” (*10; neonatal nurse*) “Yes it was a section so it was just a bit…trying to get in there and there were the surgeons there and that was more logistically a bit tricky.” (*19; SHO*) “[…] we had to cover the trolley with a sterile cover and that kind of came up over the trolley and covered the switches and that kind of thing, so I completely forgot the clock because I couldn't see it, it was completely covered over.” (*1; ANNP*) “I think one issue which I hadn't appreciated previously was the sterile versus partially sterile versus sterile nature […] and we're blurring those margins.” (*2; consultant obstetrician*) “The baby was a bit blue and it didn't yell. It was fine but because I was there with the trolley and I had a nice warm surface and a towel, during that time I was able to rub the baby, dry him, stimulate him, and so within 30 seconds he was beginning to cry and respond and that's much easier to do than with the surgeon just holding the baby and I don't know but in that situation had the trolley not been there the surgeon may have wimped out before two minutes so that we could have taken the baby over to the resuscitaire.” (*1; ANNP*)	14
4. Comparison of the trolley with usual resuscitation equipment	“No, I suppose the reason I like the old one is because on the sides they have like the side which flip out—they're like mini shelves that you can put stuff on like different size tubes and the laryngoscope, CO2 monitor and then clearly everything is next to you and you are not relying on this new method where it is a separate box and they were handing it to you. I just felt that you are more prepared when you go there because you get everything already set up.” (*12; SHO*) “It is basically just the same, it's just obviously you have got a small working area on the trolley, especially if you have got a term baby on it, there doesn't seem like much room and the sides are quite low so you feel like you have to stand next to it—well you do have to stand right next to the baby the whole time. Whereas, on the big resuscitaire you can just put the side up and walk away from it.” (*14; ANNP*)	18
5. Training and integration of bedside care into clinical routine		
Teething problems	“It's something just to get used to really rather than being a big issue. I think it's just more a case of teething problems and people on obstetrics knowing that they need to use it and where it needs to be and that kind of thing.” (*14; ANNP*) “Overall it's different, so you have to get used to it don't you?” (*25; senior paediatric registrar*) “A lot of the times we have got the delivery suite bleep. You know, you are only called a minute before the baby's out, so there is no time to go and get the trolley.” (*C8; ANNP*) “Then, because you have a CTG machine and the actual obstetrician doing the delivery, I mean, they were getting a little bit tiresome of us because they felt we were actually on top of them.” (*12; SHO*) “I think space actually, for us to get around is very good. I think being…you can see…not really you can see more, but it is more accessible because you can get all the way round.” (*15; consultant neonatologist*)	19
Training	“I would want to be with somebody experienced using the trolley” (*16; SHO*) “I did have some apprehensions when I first started using it but I think that was due to my own confidence in actually physically using it. But I think once you have done it a couple of times it is second nature and it is so easy to see all the equipment and everything.” (*9; ANNP*)	11

ANNP, advanced neonatal practitioner; number, participant number; SHO, senior house officer.

### Parents’ involvement

Eighteen clinicians mentioned that bedside care at birth allowed parents to see and touch their baby, and to see what the clinical team were doing. They felt this was especially important for babies subsequently admitted to the neonatal unit, as the parents were able to see and be with their baby before transfer. This is in contrast to usual ‘room-side care’, where the mother might not have been able to see the baby until the mother visited the neonatal unit (*4;* advanced neonatal practitioner (*ANNP*)). One clinician also felt that the baby's proximity to his/her parents made the situation “more natural” (*3; ANNP*). However, there were circumstances where the parents were not able to see or touch their baby—particularly following caesarean birth. Nine clinicians who had provided bedside care at a caesarean section stated they thought parents were not able to see their baby on the trolley because of the screen (*10; neonatal nurse*). Clinicians also explained that, in assisted vaginal births in the lithotomy position, the trolley often had to be placed close to the vulva and out of the mother's view to allow the cord to reach (if neonatal care was being given with the cord intact).

Eight clinicians reported on positive comments made by parents as a result of being close to their baby when he/she was being cared for (*12;* senior house officer (*SHO*)). This included a mother whose baby died soon after birth, who said “it was really good to be able to touch him and be close to him and spend some time with him” (*4; ANNP*). None of the clinicians mentioned negative reactions from parents when probed.

Eight clinicians commented on the impact of providing care at the bedside on their communication with parents. Four stated that they felt their proximity to the parents aided or increased their communication with them (*15; consultant neonatologist*). Three participants felt that care at the bedside made no difference in this respect, one of them stating that this was an issue of practice, not equipment, and communication with parents should not happen just “because you're near somebody's legs” (*24; ANNP*). One clinician felt that, for advanced resuscitation, a member of the staff should be assigned to support and provide explanations to the parents.

### Reservations about neonatal care at birth beside the mother

Some clinicians had concerns about the potential impact on themselves and on parents when performing certain procedures in view of parents. Of the 16 participants who spoke about the impact on clinicians, the majority had no reservations about being watched by parents, but 5 thought that staff with less experience might feel insecure being watched (*12; SHO*). One clinician wondered whether parents, after seeing their baby's treatment at birth, would expect to observe all future procedures in the neonatal unit, which might be a concern for less-experienced staff (*15; consultant neonatologist*).

Twelve clinicians commented on the impact that watching neonatal care at birth might have on parents. Five felt that it would be beneficial, while four were unsure or thought that parents might be scared, but in reality found that they were not (*15; consultant neonatologist*). Two clinicians felt that high-intensity interventions might be upsetting for parents to watch. Two clinicians suggested that parents should be asked beforehand whether they would want neonatal care at the bedside.

### Practical challenges in providing neonatal care at the bedside

Clinicians talked about circumstances where providing bedside care was more challenging, and problems encountered when using the trolley. Since the trolley was being developed as part of feasibility work in preparation for a trial evaluating deferred cord clamping, clinicians using the trolley were aiming to provide immediate newborn care with an intact umbilical cord whenever possible. Hence, instances when the cord was too short to allow this were commented on. Of the 10 participants who mentioned umbilical cord length, 7 referred to problems with placing the baby on the trolley with the cord intact (*19; SHO*), while two reported that the cord length was sufficient.

Caesarean section was another practical challenge for providing bedside care and using the trolley. Clinicians mentioned that parents were unable to see their baby during a caesarean section (see theme 1). Eight participants also commented on the need to scrub and gown, as they would be entering the sterile field. With care at the side of the room this is unnecessary, and scrubbing up took a lot of time out of their already busy work days. They also noted that some of the trolley's switches were covered by the sterile drapes, making their use awkward. One obstetrician felt there was a need for clear protocols and training because the trolley “blurred the margins” between sterile and non-sterile fields in theatre (*2; consultant obstetrician*). However, a neonatal nurse made positive comments about bedside care at a caesarean birth, reporting that she was able to dry and stimulate a “blue” baby with the cord intact, rather than having to clamp and cut the cord to transfer the baby, as would have been usual practice (*1; ANNP*).

### Comparison of the trolley with usual resuscitation equipment

Eighteen clinicians made comments comparing the trolley to the standard resuscitation equipment. The majority (10) stated no preference. Five clinicians favoured the standard equipment and three favoured the trolley. Nine clinicians mentioned that the standard equipment was used for transporting the baby to the neonatal unit, which meant the baby needed to be moved from the trolley to the standard equipment if admission to the neonatal unit was required. The main disadvantages of the trolley in comparison to the standard equipment were noted to be a smaller work surface, no space to lay out the equipment, and concern about the baby's safety on the trolley (*14; ANNP*).

### Training and integration of bedside care into clinical routine

Nineteen clinicians spoke of “teething problems” with bedside care and using the trolley (*14; ANNP*), of whom 13 mentioned the need for familiarisation and six reported that the trolley was not sufficiently integrated into hospital practice, for example, it was not routinely brought into births, and if needed at short notice, there was not always time to set it up. Most clinicians also spoke about issues related to space for staff around the trolley. The most common concern was that having the trolley by the bed interfered with other staff members’ space (*12; SHO*), especially at caesarean section. Five clinicians mentioned having had problems getting close enough to the mother, and nine clinicians stated that there was not enough space around the trolley for multiple staff members to access the baby, although some acknowledged that this problem would be alleviated by moving the trolley slightly away from the mother. Three clinicians, however, reported having good access to the baby and no problems with space around other staff (*15; consultant neonatologist)*.

Of 11 participants who mentioned issues related to training and experience, three (2 SHOs and 1 ANNP) said that they would not be comfortable using the trolley on their own, while four had gained confidence after using the trolley (*9; ANNP*). Five clinicians had not used the trolley for a full resuscitation.

## Discussion

We have previously shown that it is possible to provide immediate neonatal care at birth beside the mother, using either the mobile trolley or standard resuscitation equipment.^14^ ^16^ The findings presented here give insights into clinicians’ experiences of providing initial bedside neonatal care and resuscitation, and of using the new trolley. Most clinicians commented that providing immediate care at the bedside allowed the parents to witness and sometimes interact with their child in the first moments of his or her life. The clinicians also perceived that their close proximity to parents improved communication. Similar themes were found in interviews with parents. Parents were largely positive about bedside care as they felt it provided reassurance, and they reported feeling involved as a family.^17^

Providing immediate neonatal care at the bedside allows the umbilical cord to be left intact during initial care and resuscitation. We have shown that newborn life support can be provided with the cord intact.^19^ Developing a feasible and acceptable method to provide bedside newborn life support has allowed us to conduct a pilot trial comparing immediate with deferred (>2 min) cord clamping in very preterm births.^13^

There was some concern from clinicians, especially for the less-experienced staff, about the care they were providing being witnessed so closely by parents. These concerns echo the findings of studies that have evaluated clinicians’ opinions about family-witnessed resuscitation in other settings. There appears to be evidence that these concerns may be becoming less noticeable over time as family-witnessed resuscitation has become more widespread in clinical practice. In a study in 1987, Doyle *et al*^20^ reported that 30% of clinicians surveyed felt that they had been hampered in their activities, mainly by anxiety about being observed or by concern about possible emotional or disruptive behaviour on the part of family members. Two reviews, published in 2005,^10^ ^11^ of family-witnessed resuscitation, mostly based in adult resuscitation care, reported that clinical staff anxieties included concerns about a negative impact on performance, concerns that resuscitation efforts could be prolonged inappropriately, concerns about interference by family members and fears of increased litigation. In a recent randomised controlled trial of family presence during adult resuscitation, including 570 family members, family-witnessed resuscitation did not affect resuscitation characteristics, patient survival, or the level of emotional stress in the medical team, and did not result in more litigation. Less than 1% of the family members who witnessed the resuscitation in that study were reported to be aggressive or in conflict with the medical team.^21^ These findings are similar to those described in studies performed in children's paediatric trauma resuscitation. Two studies^22^ ^23^ reported no effect of family presence on the effectiveness or duration of the resuscitation attempt and no evidence of interference by the family on the resuscitation. The concerns expressed by clinicians in our study cannot be ignored, and institutions wishing to introduce family-witnessed resuscitation in newborn care will need to bear these concerns in mind and provide appropriate support for clinical staff. This will include education and training to embed it into clinical practice, and providing appropriate support for junior clinicians, probably including discussion within the postresuscitation team debrief recommended in national guidance (https://www.resus.org.uk/quality-standards/acute-care-quality-standards-for-cpr/#team). From the experiences reported in the adult literature, it seems likely that the negative impacts of family presence during resuscitation do not occur and, as family-witnessed resuscitation becomes embedded into practice, these staff anxieties become less marked.

One of the main themes identified was ‘training and integration of bedside care into clinical routine’. Many of the difficulties described relate to complications in changing clinical practice: having the equipment available in a timely manner, knowing where to position it, maintaining the sterile field in theatre and when to move into position. Although the bedside space is traditionally the domain of the midwifery and obstetric staff, providing bedside neonatal care requires the neonatal staff and equipment to be around the mother. This has implications for normal routines and roles, and the sequence of events at birth. None of the difficulties identified are insurmountable, but they do require a multidisciplinary team approach.

This is the first in-depth study of clinicians’ experiences of immediate newborn care and resuscitation at the maternal bedside. Use of in-depth qualitative methods allowed a detailed exploration of these experiences. Healthcare professionals with a wide range of clinical backgrounds and experience were interviewed, suggesting that the findings are representative. However, clinicians were recruited from a single site where the trolley was pioneered.

A previous questionnaire study including clinicians, about the safety, usability and acceptability of the mobile trolley, found that the clinicians felt the trolley improved parents’ overall experiences and we therefore expected to find similar findings in this study.^14^ However, the interview schedule was designed to elicit as much information as possible and non-leading interview questions were designed. Also, the interviewers were not involved in this previous study, which helped mitigate this potential bias. Throughout the process, we looked for disconfirming examples of our themes and we report these in the manuscript. We also engaged in frequent discussion of results with peers who were not part of the research team. Finally, the analysis was data driven and was led by participant responses.

## Conclusions

Most clinicians were positive about providing immediate neonatal care at the mother's bedside, particularly in terms of their perceptions of the parents’ experience. Anxieties about performing under the close scrutiny of parents were raised, similar to those raised by clinicians providing resuscitation to other patient groups. Although these have been shown to be ill founded in other areas, this merits further research and training, and support for staff in providing bedside care.

## References

[1] Lee AC, Cousens S, Wall SN Neonatal resuscitation and immediate newborn assessment and stimulation for the prevention of neonatal deaths: a systematic review, meta-analysis and Delphi estimation of mortality effect. BMC Public Health 2011;11 (Suppl 3):S12 10.1186/1471-2458-11-S3-S12 PMC3231885 21501429

[2] Arnold L, Sawyer A, Rabe H Parents’ first moments with their very preterm babies: a qualitative study. BMJ Open 2013;3:pii: e002487. 10.1136/bmjopen-2012-002487 PMC3641451 23550091

[3] Sawyer A, Rabe H, Abbott J, Very Preterm Birth Qualitative Collaborative Group. Parents’ experiences and satisfaction with care during the birth of their very preterm baby: a qualitative study. BJOG 2013;120:637–43. 10.1111/1471-0528.12104 23289929 PMC3613739

[4] Hutchon D, Burleigh A Neonatal resuscitation. AIMS J 2013;25:17.

[5] Resuscitation Council. Should relatives witness resuscitation? UK: Resuscitation Council, 1996.

[6] Critchell CD, Marik PE Should family members be present during cardiopulmonary resuscitation? A review of the literature. Am J Hosp Palliat Care 2007;24:311–17. 10.1177/1049909107304554 17895495

[7] Moons P, Norekval TM European nursing organizations stand up for family presence during cardiopulmonary resuscitation: a joint position statement. Prog Cardiovasc Nurs 2008;23:136–9. 10.1111/j.1751-7117.2008.00004.x 19039895

[8] Robinson SM, Mackenzie-Ross S, Campbell Hewson GL Psychological effect of witnessed resuscitation on bereaved relatives. Lancet 1998;352:614–17. 10.1016/S0140-6736(97)12179-1 9746023

[9] Boie ET, Moore GP, Brummett C Do parents want to be present during invasive procedures performed on their children in the emergency department? A survey of 400 parents. Ann Emerg Med 1999;34:70–4. 10.1016/S0196-0644(99)70274-X 10381997

[10] Harteveldt R Benefits and pitfalls of family presence during resuscitation. Nurs Times 2005;101:24–5. 16163929

[11] Halm MA Family presence during resuscitation: a critical review of the literature. Am J Crit Care 2005;14:494–511. 16249587

[12] Harvey ME, Pattison HM The impact of a father's presence during newborn resuscitation: a qualitative interview study with healthcare professional. BMJ Open 2013;3:e002547 10.1136/bmjopen-2013-002547 PMC3612808 23535758

[13] Pushpa-Rajah A, Bradshaw L, Dorling J Cord pilot trial—immediate versus deferred cord clamping for very preterm birth (before 32 weeks gestation): study protocol for a randomized controlled trial. BMC Trials 2014;15:258 10.1186/1745-6215-15-258 PMC4227076 24981366

[14] Thomas MR, Yoxall CW, Weeks AD Providing newborn resuscitation at the mother's bedside: assessing the safety, usability and acceptability of a mobile trolley. BMC Pediatrics 2014;14:135 10.1186/1471-2431-14-135 24885712 PMC4055396

[15] Weeks AD, Watt RJP, Hutchon DJR Innovation in immediate neonatal care: development of the Bedside Assessment, Stabilisation and Initial Cardiorespiratory Support (BASICS) trolley. 2014, http://www.nottingham.ac.uk/nctu/documents/preterm-birth/basicsdevelopment-report-29april2014.pdf 10.1136/bmjinnov-2014-000017 PMC4467574 26191414

[16] Schoonakker B, Dorling J, Oddie S Bedside resuscitation of preterm infants with cord intact is achievable using standard resuscitaire. Oporto, Portugal: European Society for Pediatric Research, 2013 Available at p430 of https://www.eiseverywhere.com/file_uploads/9206db9fe962868d47f709b38365ec5e_9349_abstract_book_-_25sett13-it-it.pdf

[18] Braun V, Clarke V Using thematic analysis in psychology. Qual Res Psychol 2006;3:77–101. 10.1191/1478088706qp063oa

[19] Duley L, Pushpa-Rajah A, Dorling J, on behalf of Cord Pilot Trial Collab Group. PC.117 Immediate versus deferred cord clamping for very preterm birth: a pilot randomised trial. Arch Dis Child Fetal Neonatal Ed 2014;99:A76–7. 10.1136/archdischild-2014-306576.218

[20] Doyle CJ, Post H, Burney RE Family participation during resuscitation: an option. Ann Emerg Med 1987;16:673–5. 10.1016/S0196-0644(87)80069-0 3578974

[21] Jabre P, Belpomme V, Azoulay E Family presence during cardiopulmonary resuscitation. New Engl J Med 2013;368:1008–18. 10.1056/NEJMoa1203366 23484827

[22] O'Connell KJ, Farah MM, Spandorfer P Family presence during pediatric trauma team activation: an assessment of a structured program. Pediatrics 2007;120:e565–74. 10.1542/peds.2006-2914 17766498

[23] Dudley NC, Hansen KW, Furnival RA The effect of family presence on the efficiency of pediatric trauma resuscitations. Ann Emerg Med 2009;53:777–84. 10.1016/j.annemergmed.2008.10.002 19013688

